# MetALD in Clinical Practice: Diagnosis, Non-Invasive Risk Stratification, and Management

**DOI:** 10.3390/jcm15197540

**Published:** 2026-09-28

**Authors:** Hubert Krzewiński, Oliwier Wątroba, Jarosław Koza

**Affiliations:** 1 Faculty of Medicine, Collegium Medicum in Bydgoszcz, Nicolaus Copernicus University in Toruń, 85-067 Bydgoszcz, Poland; 321494@stud.umk.pl; 2 Department of Gastroenterology and Nutrition Disorders, Faculty of Health Sciences, Collegium Medicum in Bydgoszcz, Nicolaus Copernicus University in Toruń, 85-168 Bydgoszcz, Poland; jaroslaw.koza@cm.umk.pl

**Keywords:** MetALD, MASLD, alcohol-related liver disease, alcohol use disorder, liver fibrosis, non-invasive tests, FIB-4, transient elastography, controlled attenuation parameter

## Abstract

Metabolic dysfunction-associated steatotic liver disease with increased alcohol consumption (MetALD) is a newly defined category within the spectrum of steatotic liver disease. It captures patients who have both cardiometabolic risk factors and alcohol intake above the thresholds accepted for metabolic dysfunction-associated steatotic liver disease (MASLD). This overlap matters clinically, as the coexistence of metabolic dysfunction and increased alcohol exposure is associated with a higher risk of progressive liver disease. In this narrative review, we bring together current evidence on the diagnosis, non-invasive risk stratification, and management of MetALD, with particular attention to serum-based fibrosis scores such as the Fibrosis-4 (FIB-4) index and imaging-based methods including vibration-controlled transient elastography and controlled attenuation parameter. We also consider the factors that can complicate the interpretation of these non-invasive tests when alcohol use is ongoing. Management has to address both components of the disease at once: reducing alcohol exposure, treating alcohol use disorder where present, managing weight, and controlling cardiometabolic risk factors. Incretin-based therapies are attracting growing interest given their established metabolic effects and the emerging hepatic and alcohol-related data, though no randomized trial has yet tested their efficacy specifically in MetALD. In advanced disease, liver transplantation remains the definitive option for appropriately selected patients, but it requires careful assessment of cardiometabolic comorbidities, alcohol-related risk, and frailty. Further prospective studies are needed to clarify the natural history of MetALD, define optimal risk-stratification strategies, and establish treatment outcomes in this population.

## 1. Introduction

### 1.1. Evolution of Nomenclature: From NAFLD/ALD to MASLD and MetALD

For decades, fatty liver disease was classified according to a rigid either/or split between Non-Alcoholic Fatty Liver Disease (NAFLD) and Alcohol-Related Liver Disease (ALD) [1,2,3]. This dichotomy drew increasing criticism, partly because it relied on exclusion criteria and partly because the term “non-alcoholic” was seen as stigmatizing. A multi-society Delphi consensus process was launched to address these limitations and to better reflect the multifactorial etiology seen in everyday practice [4]. In 2023, the American Association for the Study of Liver Diseases (AASLD), the European Association for the Study of the Liver (EASL), and the Asociación Latinoamericana para el Estudio del Hígado (ALEH) jointly introduced Steatotic Liver Disease (SLD) as an overarching term [4], and this nomenclature has since been taken up in current clinical guidelines [5].

Under this umbrella term, steatotic liver disease (SLD) now encompasses metabolic dysfunction-associated steatotic liver disease (MASLD) (Figure 1), which has replaced NAFLD and moved the diagnostic focus toward positive metabolic criteria rather than a diagnosis of exclusion [4,5]. MASLD is defined by hepatic steatosis plus at least one of five cardiometabolic criteria (Table 1). When a patient with hepatic steatosis and at least one cardiometabolic risk factor also drinks 140–350 g/week (20–50 g/day) of alcohol as a woman, or 210–420 g/week (30–60 g/day) as a man, the diagnosis becomes MetALD; above these thresholds, the ALD category applies regardless of cardiometabolic risk factor status [4,5]. Other causes of steatosis should not be forgotten, since more than one etiology can coexist, and the current nomenclature allows metabolic dysfunction plus an additional cause of steatosis to be labeled a combination etiology [4].

MetALD was thus formalized by the 2023 Delphi consensus specifically to capture the clinically important overlap between metabolic dysfunction and increased alcohol intake, an overlap that the previous NAFLD/ALD dichotomy handled poorly [4,6,7].

### 1.2. Assessment of Alcohol Exposure in MetALD

Accurate assessment of alcohol exposure matters for both diagnosis and longitudinal reclassification of MetALD. Clinicians should ask about both current and past consumption, including the quantity, frequency and pattern of drinking as well as recent periods of abstinence or substantial reduction, ideally expressing alcohol intake in grams of pure alcohol per day or week rather than in “standard drinks” alone, because the amount represented by a standard drink varies between countries and may also differ according to beverage type and serving size. Validated tools such as the Alcohol Use Disorders Identification Test (AUDIT) and its shorter AUDIT-C version are useful for structured screening, but they complement rather than replace a quantitative estimate of intake [8].

Self-reported drinking is imperfect because recall bias and underreporting are common. In selected patients, direct biomarkers can complement the clinical history: whole-blood phosphatidylethanol (PEth) reflects alcohol exposure over approximately 2–4 weeks, whereas urinary ethyl glucuronide (EtG) has a shorter detection window of about 3 days [8]. PEth reflects recent rather than historical alcohol use, may be less sensitive to low levels of drinking, and its applicability can be limited by cost [8]. These biomarkers should support, not replace, clinical history when assigning an SLD subtype. Because both alcohol exposure and cardiometabolic risk factors can change over time, classification as MASLD, MetALD, or ALD should be revisited periodically rather than treated as permanently fixed [4,8].

### 1.3. Epidemiology and Global Health Burden of MetALD

Introducing the MetALD category has reshaped the epidemiological picture of SLD, bringing into focus a group of patients the previous NAFLD/ALD dichotomy largely missed. MASLD itself is thought to affect around 38% of adults and 7–14% of children [9], and based on earlier NAFLD projections, adult prevalence could exceed 55% by 2040 [10]. Establishing the prevalence of MetALD specifically is more difficult, and estimates vary considerably across populations, partly because drinking patterns differ and partly because self-reported alcohol intake is inherently unreliable [11]. Current estimates place MetALD at roughly 2–8% of the general population [11], while a recent meta-analysis calculated a pooled prevalence of 10%, with a 95% confidence interval (CI) of 7–13%, among people with SLD [12]. Taken together, these figures identify MetALD as a clinically meaningful subgroup of SLD, although its true prevalence remains uncertain and may be underestimated.

Longitudinal observational data suggest that MetALD is associated with a higher risk of adverse liver outcomes than MASLD alone, including cirrhosis, hepatic decompensation, and hepatocellular carcinoma [13,14]. These associations should be interpreted cautiously, as differences in alcohol exposure, cardiometabolic burden, baseline fibrosis, and the limitations of self-reported alcohol intake may contribute to the observed risk estimates. This combination of metabolic dysfunction and increased alcohol intake is therefore likely to represent a substantial clinical and public health burden. As cardiometabolic risk factors become more common, early identification and non-invasive risk stratification of these patients will become increasingly important in both primary and specialist care.

### 1.4. Aims and Scope of the Review

This review brings together current evidence on MetALD, examining the utility of non-invasive biomarkers for assessing liver fibrosis alongside vibration-controlled transient elastography (VCTE), including controlled attenuation parameter (CAP) for steatosis and liver stiffness measurement (LSM) for fibrosis [5,6]. It also outlines therapeutic approaches for patients with both cardiometabolic risk factors and alcohol use disorder (AUD). The goal is practical: to provide primary care and specialist clinicians with tools that can support early risk stratification and management of MetALD.

## 2. Methodology

This narrative review draws on searches of PubMed/MEDLINE, Embase, and the Cochrane Central Register of Controlled Trials (CENTRAL) for relevant English-language publications, covering the main areas this review addresses: MetALD and MASLD nomenclature, epidemiology, pathophysiology, alcohol consumption and alcohol use disorder, non-invasive assessment of steatosis and fibrosis, and current management strategies. Since this is a narrative rather than a systematic review, the searches were iterative and topic-specific rather than following a single prespecified strategy. Representative combinations of Medical Subject Headings and free-text terms included (“steatotic liver disease” OR “MASLD” OR “MetALD” OR “NAFLD”) AND (“FIB-4” OR “APRI” OR “non-invasive assessment” OR “fibrosis” OR “steatosis”). CENTRAL was included to complement the bibliographic databases in identifying controlled intervention studies relevant to the management sections of the review.

We considered original studies, systematic reviews and meta-analyses, clinical practice guidelines, consensus documents, and selected narrative reviews whenever they were directly relevant to the clinical questions at hand. When several types of evidence were available, current clinical practice guidelines, consensus statements, systematic reviews and meta-analyses were generally prioritized, supplemented by key original studies where necessary. The clinical evidence synthesis focused primarily on adult populations. Pediatric data were not systematically reviewed; however, age-specific consensus diagnostic criteria and limited epidemiological data were retained where necessary to present the current SLD nomenclature and its broader context. Non-English publications, conference abstracts without full-text publication, letters, editorials, and animal studies were excluded. Older publications were included where needed to trace the derivation of established diagnostic scores or thresholds, and major international hepatology guidelines and consensus statements were reviewed as well. The principal electronic literature search covered January 2006 through August 2026.

Literature screening, including title, abstract, and full-text assessment, and final source selection were independently conducted by O.W., H.K., and J.K., with disagreements resolved through consensus.

Generative AI tools supported preliminary literature searching, source identification, language and readability editing, manuscript revision, and the preparation of figures and tables. Every AI-assisted output and suggested source was independently reviewed by the authors, and all citations and factual statements the AI contributed to were checked against the original sources before being included in the manuscript.

## 3. Pathophysiological Interplay

The pathophysiology of MetALD is less a linear chain of events than a set of alcohol-related and metabolic mechanisms acting at the same time [6,15,16]. Ethanol exposure operates alongside insulin resistance, adipose tissue dysfunction, gut dysbiosis, oxidative stress, and lipotoxicity, and these processes tend to reinforce one another, driving hepatic inflammation and fibrogenesis forward.

### 3.1. Alcohol Component

Most alcohol metabolism proceeds through oxidative pathways, with non-oxidative routes playing a smaller role. In the cytosol, alcohol dehydrogenase converts ethanol into acetaldehyde, which mitochondrial aldehyde dehydrogenase 2 then oxidizes to acetate. This raises the NADH/NAD+ ratio, which in turn impairs fatty acid oxidation and promotes hepatic triglyceride accumulation and steatosis [17,18]. With chronic exposure, the microsomal ethanol-oxidizing system, driven mainly by cytochrome P450 2E1, becomes more active, generating reactive oxygen species (ROS), lipid peroxidation, and mitochondrial injury [17,19]. Non-oxidative metabolism produces fatty acid ethyl esters, ethyl glucuronide (EtG), ethyl sulfate (EtS), and phosphatidylethanol (PEth). EtG, EtS, and PEth are particularly useful as direct biomarkers of alcohol exposure, while fatty acid ethyl esters have also been linked to direct cellular and mitochondrial toxicity [20,21].

Beyond direct oxidative injury, ethanol and acetaldehyde also affect the gut. They weaken the intestinal epithelial barrier by disrupting tight junction proteins such as occludin and zonula occludens-1, contributing to increased intestinal permeability [22,23]. Alcohol exposure also alters the composition of the intestinal microbiome, both qualitatively and quantitatively. Once the barrier is compromised, pathogen-associated molecular patterns (PAMPs), most notably Gram-negative lipopolysaccharide (LPS), can translocate into the portal circulation. When LPS reaches the liver, it activates Toll-like receptor 4 (TLR4) signaling, particularly in Kupffer cells, and downstream nuclear factor kappa B (NF-κB) activation promotes the release of pro-inflammatory mediators such as tumor necrosis factor alpha (TNF-α), adding to hepatic inflammation [22,23,24].

### 3.2. Metabolic Component

The metabolic component of the disease is driven largely by systemic insulin resistance and the lipotoxicity that follows. Under physiological conditions, insulin suppresses lipolysis in white adipose tissue; once insulin resistance develops, this inhibitory effect weakens and more non-esterified free fatty acids (FFAs) enter the portal and systemic circulation [25,26]. At the same time, hyperinsulinemia stimulates hepatic lipogenesis via sterol regulatory element-binding protein 1c, while greater carbohydrate availability activates carbohydrate-responsive element-binding protein. Together, these pathways increase de novo lipogenesis (DNL) [27,28,29].

Once the combined burden of circulating FFAs and DNL-derived lipids exceeds the liver’s capacity to oxidize or export them, lipotoxic intermediates such as diacylglycerols, ceramides, and lysophosphatidylcholine accumulate. These molecules promote mitochondrial dysfunction, ROS generation, and endoplasmic reticulum stress, eventually leading to hepatocyte injury and death [30]. Damage-associated molecular patterns released in the process, together with ongoing oxidative and inflammatory signaling, activate hepatic stellate cells and promote their transformation into collagen-producing myofibroblasts, driving progressive hepatic fibrogenesis [31].

### 3.3. Host and Lifestyle Modifiers

Host and lifestyle factors also shape susceptibility to liver injury in MetALD. Genetic variants in PNPLA3, TM6SF2, and HSD17B13 have been linked to differences in susceptibility to steatosis, fibrosis progression, cirrhosis, and liver-related complications across metabolic and alcohol-related liver disease more broadly. Data specific to MetALD remain limited, but recent studies suggest that these variants may also influence disease progression in this population [32].

Sex is another important modifier: women can develop advanced alcohol-related liver disease at lower levels of alcohol exposure than men, which is reflected in the sex-specific alcohol thresholds used in the current MetALD definition [33]. Smoking is a further modifiable risk factor and has been linked to worse clinical outcomes in patients with SLD, including MetALD [34]. Together, these factors may contribute to the substantial variability seen between patients with otherwise similar metabolic and alcohol exposure profiles.

## 4. Non-Invasive Serum Biomarkers for Fibrosis Assessment

### 4.1. First-Line Triage: FIB-4 Index and APRI

The degree of hepatic fibrosis is among the strongest predictors of prognosis in SLD, particularly for liver-related outcomes and mortality [35]. Simple serum-based fibrosis scores have therefore become useful first-line risk-stratification tools because they are inexpensive, widely available, and non-invasive [36].

FIB-4 (the Fibrosis-4 index) is the score most widely recommended as a first-line triage tool for patients at risk of advanced fibrosis [5,37]. It draws on age, aspartate aminotransferase (AST), alanine aminotransferase (ALT), and platelet count. APRI (AST-to-Platelet Ratio Index) is another readily available option, although it appears to perform less well in SLD, including MetALD [38,39]. Both are calculated as shown in Figure 2.

A FIB-4 below 1.30 generally indicates a low probability of advanced fibrosis; values between 1.30 and 2.67 fall into an intermediate-risk range; and above 2.67, the probability of advanced fibrosis increases [5,37,38]. In patients aged 65 years or older, the lower cut-off is raised from 1.3 to 2.0 because specificity declines with age [5,37]. Importantly, these thresholds were not derived specifically in patients with MetALD and should be treated as risk-stratification tools rather than histological diagnostic boundaries.

Nevertheless, recent MetALD-specific studies support the clinical value of FIB-4. Against histological or magnetic resonance elastography reference standards, FIB-4 showed clinically useful discrimination for advanced fibrosis, while APRI generally performed less well [38,39,40]. Roy et al. evaluated 168 patients with MetALD in a retrospective analysis of a prospectively maintained liver-clinic database; 31/168 (18.4%) had MRE-defined advanced fibrosis (MRE ≥ 3.53 kPa) [38]. Gu et al. conducted a multinational, multicenter, retrospective cross-sectional study; among 369 patients with MetALD, the biopsy-proven subgroup included 192 patients, of whom 44 (22.9%) had advanced fibrosis (≥F3) [39]. Torp et al. evaluated 102 patients with MetALD from two prospectively recruited cohorts; liver histology was available in 78 patients, of whom 19 (24%) had advanced fibrosis (≥F3) [40]. These studies represent subsequent evaluation of established thresholds in MetALD rather than derivation of a new, disease-specific score.

**Figure 2:**
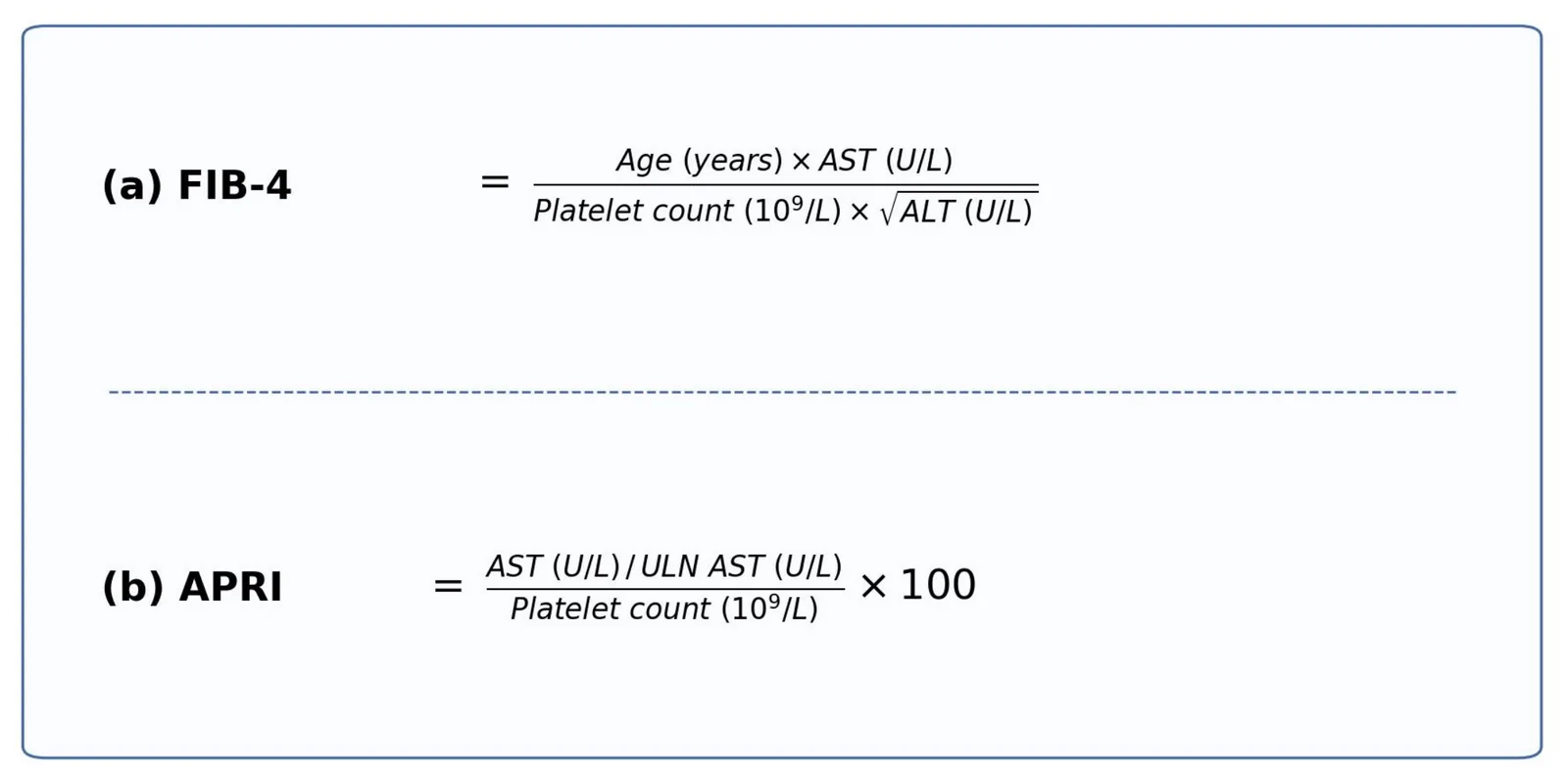
Equations for calculating (**a**) the Fibrosis-4 (FIB-4) index and (**b**) the AST-to-Platelet Ratio Index (APRI). The equations were originally described by Sterling et al. [41] and Wai et al. [42], respectively; the graphic was prepared by the authors. Abbreviations: ALT, alanine aminotransferase; APRI, AST-to-Platelet Ratio Index; AST, aspartate aminotransferase; FIB-4, Fibrosis-4 index; ULN, upper limit of normal.

### 4.2. Advanced Commercial Panels: ELF Test and FibroTest

When first-line scores are inconclusive, more advanced serum tests can add useful information. The Enhanced Liver Fibrosis (ELF) (Siemens Healthineers, Erlangen, Germany) test is a direct panel based on markers of extracellular matrix turnover, while FibroTest (BioPredictive, Paris, France) combines indirect biochemical markers associated with fibrosis [43].

ELF measures hyaluronic acid, tissue inhibitor of metalloproteinases 1, and amino-terminal propeptide of type III procollagen [43]. The combined score has been validated for detecting advanced fibrosis and also carries prognostic value in chronic liver disease more generally [44,45]. A value below 9.8 is typically used to rule out advanced fibrosis, and 11.3 or above to rule it in, although neither cut-off was originally derived specifically for MetALD.

MetALD-specific data support the diagnostic utility of ELF. In the Torp et al. [40] cohort, liver histology was available in 78 patients with MetALD, including 19 with advanced fibrosis (≥F3). An ELF below 9.8 had a sensitivity of 100% (95% CI 82–100%) and a negative predictive value of 100% (95% CI 91–100%) for advanced fibrosis, while an ELF of 11.3 or above reached a specificity of 93% (95% CI 84–98%) [40]. These findings support ELF as a potential second-line option, although further external validation specifically in MetALD is still needed.

FibroTest combines haptoglobin, alpha-2-macroglobulin, total bilirubin, apolipoprotein A1, and gamma-glutamyl transferase, adjusted for age and sex, into a score that correlates with hepatic fibrosis [43]. It has been studied across several chronic liver diseases, including alcohol-related and metabolic liver disease, but evidence specific to MetALD remains limited.

ELF can be especially useful when FIB-4 is elevated or indeterminate and elastography is not readily accessible. In the Torp et al. MetALD cohort, using ELF before transient elastography maintained diagnostic performance while reducing referrals for elastography by 43%, corresponding to 20 of 47 patients who would otherwise have undergone transient elastography [40].

### 4.3. Limitations in the Context of Active Alcohol Consumption

Ongoing alcohol use can complicate the interpretation of indirect serum fibrosis scores, particularly FIB-4 and APRI, because both rely on laboratory values that can change for reasons unrelated to fibrosis. Alcohol-related inflammation can raise aminotransferase levels, and heavy drinking can also lower platelet counts [46,47,48]. An elevated FIB-4 or APRI in a patient who is actively drinking should therefore be interpreted alongside current alcohol exposure and biochemical evidence of hepatic inflammation.

These limitations do not preclude the use of FIB-4 in alcohol-related or metabolic-alcohol-related liver disease, but an elevated result should not be interpreted in isolation. If recent alcohol exposure coincides with biochemical evidence of inflammation, reassessment after the patient reduces or stops drinking may help clarify the result, particularly when combined with a second-line test [49,50].

## 5. Imaging-Based Non-Invasive Tests

Serum-based scores such as FIB-4 are useful for identifying patients with a low probability of advanced fibrosis, but their accuracy is more limited outside that low-risk range. Patients above the low-risk threshold generally need a second-line test, and vibration-controlled transient elastography (VCTE) is one of the most widely used imaging-based options for this purpose [5,37].

### 5.1. Vibration-Controlled Transient Elastography: Clinical Role in Fibrosis Risk Assessment

VCTE is a well-established second-line, non-invasive tool for assessing fibrosis risk [5,50]. It is particularly useful after an elevated or indeterminate first-line serum test and can reduce the need for liver biopsy in routine clinical practice.

The technique works by using a probe to generate low-frequency mechanical vibrations, which send a shear wave through the liver. Pulse-echo ultrasound then tracks the speed of that wave, and the result is converted into a liver stiffness measurement (LSM), expressed in kilopascals [51,52].

Technical quality is essential because inadequate acquisition can compromise the reliability of the result. Patients should fast for at least 3 h before the examination [50], and a valid reading conventionally requires at least 10 successful measurements. Both the median LSM and the interquartile range-to-median ratio (IQR/M) should be reported; an IQR/M of 0.30 or below has traditionally been considered acceptable, although reliability also depends on the absolute LSM value. An IQR/M above 0.30 is more concerning when the median LSM is 7.1 kPa or higher [53]. Probe choice should follow device recommendations and skin-to-liver capsule distance. EASL reports applicability above 95% when the appropriate probe is used in patients who are not morbidly obese, and in NAFLD cohorts, using both M and XL probes brings acquisition failure below 5% [50]. In the cohort studied by Boursier et al., 24.3% of examinations were unreliable by the traditional criteria, but only 9.1% were classified as poorly reliable under the revised, IQR/M- and LSM-dependent definition [53].

LSM should be viewed as a continuous marker of liver stiffness rather than a direct equivalent of histological fibrosis stage. The thresholds commonly used in alcohol-related or metabolic liver disease were not derived specifically for MetALD. Values under 8 kPa generally indicate a low probability of advanced fibrosis, while values around 12–15 kPa or higher indicate a higher probability once confounders have been considered [50]. Recent MetALD-specific studies support the usefulness of VCTE and sequential FIB-4–VCTE pathways, but they do not establish a separate, MetALD-specific set of cut-offs [39,40].

The Baveno VII criteria use a related but separate framework for compensated advanced chronic liver disease (cACLD): an LSM below 10 kPa, in the absence of other clinical or imaging signs, makes cACLD unlikely; 10–15 kPa is suggestive; and above 15 kPa is highly suggestive [54].

For patients with cACLD, combining LSM with platelet count allows non-invasive assessment of clinically significant portal hypertension (CSPH). CSPH can be ruled out when LSM is 15 kPa or below and platelet count is 150 × 10^9^/L or higher. An LSM of 25 kPa or above can rule in CSPH in virus- and/or alcohol-related cACLD and in non-obese cACLD related to non-alcoholic steatohepatitis (NASH), although performance is lower in patients with obesity [54].

Whether to perform screening endoscopy is a separate question from diagnosing CSPH. In patients with compensated cirrhosis who cannot take non-selective beta-blockers because of contraindication or intolerance, endoscopy is indicated when LSM is 20 kPa or higher or platelet count is 150 × 10^9^/L or below. If LSM is under 20 kPa and platelet count is above 150 × 10^9^/L, endoscopy can be deferred in favor of continued non-invasive follow-up [54]. In patients who already qualify for non-selective beta-blocker therapy, endoscopy is not required solely to decide whether to start primary prophylaxis (Table 2).

### 5.2. Controlled Attenuation Parameter for Steatosis

Controlled attenuation parameter (CAP), measured alongside VCTE, quantifies the ultrasound attenuation caused by hepatic fat accumulation and is expressed in decibels per meter (dB/m) [55].

Conventional ultrasound remains the most widely accessible method for detecting hepatic steatosis, although it is less sensitive for mild disease and depends partly on image quality and operator interpretation. A meta-analysis of 49 studies and 4720 participants found a sensitivity of 84.8% and specificity of 93.6% for detecting moderate-to-severe steatosis against histology [56].

The commonly cited CAP thresholds, roughly 248, 268, and 280 dB/m for ≥S1, ≥S2, and S3 steatosis, come from an individual-patient-data meta-analysis of mixed chronic liver disease populations [55]. Most participants had viral liver disease, with only a minority having NAFLD/NASH, and none of the thresholds were derived specifically for MetALD. They should therefore be interpreted as approximate, probabilistic cut-offs rather than precise histological boundaries. Because the confidence intervals around the proposed ≥S2 and S3 thresholds overlap substantially, CAP cannot reliably distinguish between these steatosis grades in an individual patient.

CAP performance can also be affected by BMI, diabetes, and the underlying cause of liver disease [55]. CAP measures steatosis but should not be used by itself to stage fibrosis or assign cardiovascular or metabolic risk. No specific CAP value has been established as a therapeutic target, and reduction below 248 dB/m is not, in itself, a required treatment goal.

MRI proton density fat fraction (MRI-PDFF) provides a more precise quantitative assessment of hepatic fat and is generally regarded as a non-invasive reference standard, although cost and limited availability restrict routine use [57]. In patients with obesity, the XL probe can improve VCTE/CAP feasibility; probe choice should follow device recommendations and skin-to-liver capsule distance rather than BMI alone.

### 5.3. Impact of Active Alcohol Use and Hepatic Inflammation

LSM reflects liver stiffness as a physical property and is not a direct measure of collagen content. Values can therefore increase not only because of fibrosis but also because of hepatic inflammation, extrahepatic cholestasis, or hepatic congestion [50,58].

This is particularly relevant in patients with recent or ongoing heavy alcohol consumption, because alcohol-related inflammation can transiently increase LSM and lead to overestimation of fibrosis [58]. Clinicians should consider elevated AST or gamma-glutamyl transferase (GGT), especially values above roughly twice the upper limit of normal, when interpreting VCTE in these patients [50].

If elevated LSM is accompanied by biochemical evidence of alcohol-related inflammation, current EASL guidance recommends repeating the test after at least one week of abstinence or a substantial reduction in drinking [50]. The timing of reassessment should be considered in the clinical context and in relation to the evolution of biochemical evidence of inflammation. This does not reflect rapid regression of fibrosis; rather, the inflammatory component is dynamic and may improve quickly after alcohol exposure is reduced.

Prospective data from Legros et al. illustrate this point: in a study of 259 patients, among those with an initial LSM of 10–25 kPa who ultimately did not have cACLD, LSM fell below 10 kPa in 44% after one month of alcohol withdrawal and in 68% after two months [59]. A separate retrospective study of 37 patients by Bardou-Jacquet et al. found that liver stiffness fell in 85% of the 13 abstinent patients over a median follow-up of 32.5 weeks, by a median of 4.9 kPa, while the 24 patients who relapsed showed no significant overall change [60]. Together, these findings support cautious interpretation of a single elevated VCTE result obtained during active drinking.

### 5.4. Alternative Imaging Modalities: 2D-SWE and MRE

Two-dimensional shear wave elastography (2D-SWE) offers an alternative ultrasound-based method for assessing liver stiffness, with the advantage of real-time B-mode visualization that allows the operator to select the measurement area [51,52].

Magnetic resonance elastography (MRE) is highly accurate for non-invasive liver stiffness assessment and samples a substantially larger volume of liver tissue than ultrasound-based methods, reducing sampling variability [61,62]. It is also less affected by obesity than VCTE and can often provide reliable measurements when ultrasound-based elastography is technically limited.

Important limitations include higher cost, longer scan times, limited availability, severe claustrophobia, MRI-incompatible implants, and body dimensions that exceed scanner capacity. Marked hepatic iron overload can degrade conventional gradient-recalled-echo MRE through signal loss, although newer sequences may partly overcome this limitation. Ascites is not an absolute contraindication, although massive ascites increases the risk of technical failure with conventional gradient-recalled-echo sequences [63]. MRE may therefore be particularly useful when other non-invasive tests are inconclusive or discordant, or when reliable ultrasound-based elastography cannot be obtained (Table 3).

## 6. Clinical Management and Risk Stratification Algorithms

### 6.1. Sequential Diagnostic Pathways

The number of patients with metabolic risk factors and increased alcohol intake makes universal referral to hepatology neither necessary nor feasible. Sequential non-invasive testing offers a practical approach to fibrosis risk stratification. FIB-4, calculated from routine clinical and laboratory data, is recommended as a first-line triage tool because of its strong negative predictive value for advanced fibrosis [5,37].

Patients with a FIB-4 below 1.30, or below 2.0 if they are 65 years or older, can generally be managed without immediate hepatology referral, provided their cardiometabolic risk factors and alcohol exposure continue to be monitored and reassessed periodically [5,37]. Those above the age-appropriate threshold should undergo second-line testing, usually with VCTE, ELF, or another validated non-invasive test. An elevated FIB-4 on its own does not establish advanced fibrosis and does not automatically require hepatology referral.

Patients whose second-line results indicate a high probability of advanced fibrosis or cACLD should be referred for specialist evaluation and, where appropriate, further assessment for cirrhosis and portal hypertension. Sequential pathways can substantially reduce unnecessary specialist referrals while maintaining identification of patients at higher risk of advanced liver disease [66,67]. Much of the prospective evidence supporting this approach, however, comes from NAFLD/MASLD populations rather than MetALD specifically. Recent MetALD-specific studies support the diagnostic and prognostic value of these established sequential strategies in this population [39,40].

In selected patients, more complex composite scores can add further information. The FibroScan-AST (FAST) score combines LSM, CAP, and AST to identify patients with a higher probability of fibrotic steatohepatitis, while Agile 3+ and Agile 4 combine VCTE with clinical and laboratory variables to estimate the probability of advanced fibrosis and cirrhosis, respectively [64,65]. These scores were developed and validated mainly in MASLD populations, and their optimal thresholds and real-world performance in MetALD are not yet well established.

Neither FIB-4 nor APRI should be treated as a short-term measure of treatment response. Alcohol reduction, weight loss, and treatment of metabolic disease can change aminotransferase levels and platelet counts well before substantial changes in hepatic fibrosis occur. Changes in these scores during follow-up should therefore be interpreted in the broader clinical context [68].

Once cirrhosis is established, patients should enter standard surveillance and complication-prevention pathways, including assessment for portal hypertension and regular hepatocellular carcinoma (HCC) surveillance. Current guidance recommends liver ultrasound roughly every 6 months for cirrhotic patients who would be candidates for HCC treatment [69]. Evidence remains insufficient to justify routine HCC surveillance solely on the basis of non-cirrhotic MetALD (Figure 3).

### 6.2. Management of Alcohol Use Disorder in MetALD

Reduction in alcohol exposure is central to MetALD management, and any coexisting alcohol use disorder (AUD) should be identified and treated as part of the overall liver disease strategy. In patients without advanced fibrosis or cirrhosis, current guidance recommends discouraging alcohol consumption but does not define a separate MetALD-specific numerical target for alcohol intake [5]. Complete, sustained abstinence is recommended for patients with advanced fibrosis or cirrhosis [5]. Treatment should combine behavioral interventions with pharmacotherapy where appropriate and should be tailored to liver disease severity, renal function, other medications, neuropsychiatric comorbidities, and patient preferences [70,71].

Because SLD categories reflect current alcohol exposure, a patient whose intake falls below the MetALD range may meet the current definition of MASLD if hepatic steatosis and cardiometabolic criteria persist [4,8]. Such reclassification should not be interpreted as resolution of previous alcohol-related risk or fibrosis, nor should it by itself determine a different follow-up interval. Follow-up should remain guided by fibrosis stage, cardiometabolic risk, and longitudinal alcohol exposure [5,8]. Serial non-invasive tests should likewise be interpreted in the context of changing alcohol exposure and biochemical activity.

Baclofen is among the best-studied pharmacological options for AUD in patients with cirrhosis. It undergoes little hepatic metabolism and is cleared mainly by the kidneys, but sedation and neurocognitive adverse effects can be clinically important, particularly in patients at risk of hepatic encephalopathy. Dose adjustment and close monitoring are needed in renal impairment [70,71,72].

Acamprosate similarly undergoes minimal hepatic metabolism, making it an attractive option in liver disease. Because it is primarily eliminated by the kidneys, renal function should be assessed before treatment; the dose should be reduced in moderate renal impairment, and the drug is contraindicated in severe renal impairment [71].

Naltrexone is effective for AUD and can be considered in compensated liver disease. Historical concerns about hepatotoxicity have limited its use, but more recent observational data in patients with cirrhosis are reassuring. Evidence for routine use in decompensated disease remains limited, and hepatic function should be considered during treatment [71,73]. Naltrexone is also contraindicated in patients receiving opioids or with current opioid dependence.

Gabapentin and topiramate may also be considered in selected patients, although the evidence base in advanced liver disease is less extensive. Both require careful attention to renal function, and sedation or cognitive adverse effects can limit their use in patients at risk of hepatic encephalopathy. Disulfiram should generally be avoided in liver disease because of the risk of hepatotoxicity [71]. Importantly, the evidence for these strategies derives mostly from AUD and alcohol-related liver disease studies rather than MetALD-specific trials (Table 4).

### 6.3. Interventions for the Metabolic Component

Lifestyle intervention remains the cornerstone of managing the metabolic component of MetALD. A sustained calorie deficit, ideally through an individualized Mediterranean-style diet combined with regular physical activity, should be encouraged. Weight loss of 5% or more is associated with reduced liver fat, 7–10% with improved liver inflammation, and 10% or more with a greater likelihood of fibrosis improvement [5,74]. Achieving and maintaining these targets through lifestyle changes alone remains difficult for many patients.

These lifestyle and weight-loss targets are derived primarily from evidence in MASLD and metabolic dysfunction-associated steatohepatitis (MASH), together with current SLD guidance, rather than from intervention trials conducted specifically in MetALD.

Incretin-based therapies have considerably expanded the pharmacological options for obesity and metabolic disease and have also shown clinically meaningful hepatic effects in MASH. In the phase 3 ESSENCE trial, 1197 patients with biopsy-confirmed MASH and F2–F3 fibrosis were randomized, with a prespecified interim analysis covering the first 800 participants who completed 72 weeks of treatment. MASH resolved without fibrosis worsening in 62.9% of patients receiving semaglutide versus 34.3% receiving placebo, and fibrosis improved without MASH worsening in 36.8% versus 22.4%, respectively [75].

Similar findings were reported in the phase 2 SYNERGY-NASH trial, in which 190 patients with MASH and F2–F3 fibrosis were randomized to tirzepatide or placebo for 52 weeks. MASH resolution without fibrosis worsening occurred in 44%, 56%, and 62% of patients receiving tirzepatide 5, 10, and 15 mg, respectively, versus 10% with placebo. Fibrosis improvement without worsening of MASH occurred in 55%, 51%, and 51% of patients receiving tirzepatide 5, 10, and 15 mg, respectively, versus 30% with placebo [76]. The trial was not designed to demonstrate a long-term clinical outcome benefit.

Retatrutide, a triple agonist acting on receptors for glucose-dependent insulinotropic polypeptide (GIP), glucagon-like peptide-1 (GLP-1), and glucagon, represents a further investigational approach. In a phase 2a MASLD substudy of 98 participants, the 12 mg dose reduced liver fat on MRI-PDFF by 82.4% at 24 weeks, compared with a 0.3% increase with placebo, for an estimated treatment difference of −82.7% (95% CI −95.2 to −70.2; *p* < 0.001) [77]. The reduction remained substantial at 48 weeks, although fewer participants had MRI data by then, and the study did not include histological assessment of MASH or fibrosis.

Separate randomized trials have examined whether GLP-1 receptor agonism can also influence alcohol-related outcomes. A 9-week phase 2 trial in 48 adults with AUD found that low-dose semaglutide reduced laboratory-measured alcohol consumption, drinks per drinking day, and alcohol craving compared with placebo, although not every drinking-related outcome improved [78]. More recently, a 26-week trial in 108 treatment-seeking patients with AUD and obesity showed that semaglutide 2.4 mg plus cognitive behavioral therapy reduced heavy drinking days more than placebo plus the same behavioral therapy [79].

Importantly, the hepatic trials and the AUD trials enrolled different patient populations. No randomized trial has yet tested the combined hepatic, metabolic, and alcohol-related efficacy of incretin-based therapy specifically in MetALD. The current evidence should therefore be regarded as indirect support for potential relevance rather than proof of efficacy in MetALD itself.

Safety in advanced liver disease also deserves attention. A randomized phase 2 trial in patients with compensated MASH-related cirrhosis found that semaglutide did not significantly improve fibrosis or MASH resolution over 48 weeks, although no hepatic decompensation events or deaths occurred and hepatic and renal function remained stable [80]. Evidence in decompensated cirrhosis is still lacking.

Substantial drug-induced weight loss may also be accompanied by loss of lean mass. The SURMOUNT-1 body-composition substudy found that tirzepatide reduced both fat and lean mass considerably [81]. These findings do not establish drug-induced sarcopenia, but they underline the importance of preserving muscle mass through adequate nutrition and physical activity, especially in patients with advanced chronic liver disease (Table 5).

### 6.4. Liver Transplantation in Advanced MetALD

Although indications for liver transplantation (LT) in MetALD do not fundamentally differ from those in other causes of advanced chronic liver disease, transplant evaluation may be more complex because advanced liver disease often coexists with substantial cardiometabolic and alcohol-related risk. In a US UNOS registry analysis, MetALD accounted for 16.2% of waitlist registrations and 15.9% of liver transplants by 2022, having become the third most common etiology in both settings since 2020 [82]. Obesity, diabetes, cardiovascular disease, renal dysfunction, and frailty or sarcopenia may therefore influence transplant evaluation and long-term risk [83,84,85]. Standard indications for LT, including decompensated cirrhosis and selected liver malignancies, and Model for End-Stage Liver Disease (MELD)-based allocation principles remain applicable [84].

Renal dysfunction is particularly relevant during transplant assessment. Acute kidney injury may have several causes, including hepatorenal syndrome–acute kidney injury (HRS-AKI), which occurs in patients with advanced cirrhosis and ascites and requires evaluation for alternative or coexisting causes of kidney dysfunction [86]. Its severity, duration, and reversibility are important considerations in transplant planning.

Cardiovascular assessment is particularly important given the cardiometabolic burden of MetALD, with further evaluation for coronary artery disease guided by individual risk. Hepatopulmonary syndrome and portopulmonary hypertension should also be considered according to standard transplant-evaluation principles [87,88].

Assessment of alcohol use should be integrated into transplant evaluation. Contemporary practice has shifted away from a rigid 6-month abstinence rule toward individualized psychosocial assessment, relapse-risk evaluation, and engagement with AUD treatment [84,88]. Structured tools such as the Stanford Integrated Psychosocial Assessment for Transplantation can support this evaluation [89]. Direct biomarkers, particularly PEth, can complement clinical and psychosocial assessment before and after transplant. A recent blood transfusion should be considered when interpreting a positive PEth result, because transfused red blood cells can transiently raise PEth levels [90].

Post-transplant evidence specific to MetALD remains limited. Registry data suggest differences in selected outcomes compared with ALD, and recent transplant-evaluation studies also point to differences in cardiometabolic comorbidity burden and recompensation. These findings should not be interpreted as proof that the MetALD diagnosis itself, independent of other factors, determines post-transplant prognosis [82,85].

Long-term care should address both components of the disease. Immunosuppressive therapy can worsen hypertension, diabetes, dyslipidemia, and weight gain, while recurrent alcohol exposure or recurrent steatotic liver disease can damage the graft [91,92]. Multidisciplinary follow-up involving transplant hepatology, cardiology, metabolic care, addiction medicine, and transplant surgery is therefore particularly important.

## 7. Discussion

Formal recognition of MetALD as a distinct category within SLD underscores the clinical importance of metabolic dysfunction and increased alcohol intake occurring together. Current observational evidence suggests an association between MetALD and a greater risk of adverse liver outcomes than MASLD alone, although the natural history and rate of fibrosis progression in this newly defined group remain incompletely characterized [13,14].

The growing availability of non-invasive tests has allowed a shift away from biopsy-based assessment toward sequential risk-stratification pathways. FIB-4 is useful as a first-line tool for identifying patients with a low probability of advanced fibrosis, and VCTE, ELF, and other second-line tests can then stratify patients above that low-risk threshold [5,37,39,40]. Importantly, most thresholds in routine use were originally developed in MASLD, ALD, viral hepatitis, or mixed chronic liver disease populations. Several have since been evaluated in MetALD cohorts, but their use in MetALD should remain probabilistic and clinically contextualized rather than be treated as fixed, disease-specific diagnostic boundaries.

Sequential testing can reduce unnecessary specialist referrals and concentrate further assessment on patients at higher risk of advanced liver disease. Much of the prospective evidence supporting this approach, however, comes from MASLD or other chronic liver disease populations rather than MetALD. Recent MetALD-specific studies provide encouraging support, but dedicated prospective validation and longer-term outcome data remain limited [39,40].

Clinical management of MetALD requires simultaneous attention to alcohol exposure and metabolic dysfunction. Reducing alcohol consumption is a central goal, and complete, sustained abstinence is recommended once fibrosis is advanced or cirrhosis has developed [5,70]. Weight management and treatment of diabetes, dyslipidemia, hypertension, and cardiovascular risk are equally important.

Incretin-based therapies are of particular interest because randomized trials have shown metabolic and hepatic benefits in MASH, while separate AUD trials suggest effects on selected alcohol-related outcomes [75,76,78,79]. However, these effects were demonstrated in different patient populations, and no trial has yet established the combined hepatic, metabolic, and alcohol-related efficacy or safety of these therapies specifically in MetALD. The potential for lean-mass loss during substantial weight reduction and the limited evidence in advanced or decompensated liver disease warrant additional caution.

The gut–liver axis represents another potential target, although the clinical evidence remains preliminary. Small randomized studies have tested fecal microbiota transplantation (FMT) in alcohol-associated cirrhosis with AUD and in MASLD, with mixed results for alcohol-related and hepatic outcomes [93,94]. No trial has evaluated FMT specifically in MetALD, so microbiota-directed interventions should currently be regarded as experimental rather than established treatment.

In advanced disease, patients undergoing transplant evaluation may present with complications of cirrhosis together with substantial cardiometabolic, renal, and alcohol-related risk. This requires careful cardiovascular, metabolic, renal, psychosocial, and addiction assessment before transplant and continued multidisciplinary follow-up afterward [84,88]. MetALD-specific transplant data remain limited, although differences in comorbidity burden, recompensation, and selected waitlist or post-transplant outcomes have been reported [82,85]. These findings should not be interpreted as evidence that MetALD itself independently determines transplant prognosis.

Several limitations of the current evidence base deserve emphasis. MetALD was recognized only recently as a distinct SLD category, and much of the evidence relevant to its diagnosis and management was generated in populations labeled NAFLD, MASLD, ALD, or other chronic liver diseases [4,5]. Such evidence can still guide practice, but it is not equivalent to direct MetALD-specific validation. Assessment of alcohol exposure adds another challenge because drinking patterns can change over time and self-report is prone to recall bias and underreporting. Prospective MetALD-specific evidence also remains limited for the long-term performance of non-invasive tests, pharmacological treatment, and liver transplantation [39,40,85]. Future research should prioritize prospective MetALD cohorts and intervention studies that track liver-related, metabolic, and alcohol-related outcomes together rather than in isolation.

Despite these limitations, the available evidence supports an integrated approach to MetALD based on accurate assessment of alcohol exposure, sequential non-invasive risk stratification, management of cardiometabolic disease, and appropriate intervention for harmful drinking or AUD. For patients with more advanced or complex disease, multidisciplinary care involving primary care, hepatology, metabolic medicine, cardiology, addiction medicine, and, where needed, transplant services is likely to be especially valuable [5,37].

## 8. Conclusions

MetALD represents a clinically important overlap between metabolic dysfunction and increased alcohol consumption, and its management requires assessment of both components of the disease. Sequential non-invasive risk stratification, starting with a simple serum-based test such as FIB-4 and moving to VCTE, ELF, or other second-line methods when needed, offers a practical way to identify patients at risk of advanced liver disease.

Management should combine reduced alcohol exposure with treatment of obesity, diabetes, dyslipidemia, hypertension, and other cardiometabolic risk factors. In patients with AUD, structured behavioral and pharmacological treatment should be considered, and complete, sustained abstinence is particularly important once fibrosis is advanced or cirrhosis has developed. Incretin-based therapies have shown metabolic and hepatic benefits in MASH and may affect selected alcohol-related outcomes in AUD, but their efficacy and safety in MetALD specifically remain to be established.

For patients with decompensated cirrhosis or other accepted indications, liver transplantation remains the definitive treatment and requires an integrated assessment of cardiometabolic, renal, psychosocial, and alcohol-related risk.

Further prospective studies are needed to better define the natural history of this newly recognized entity, along with optimal risk-stratification strategies, treatment response, and long-term outcomes.

## Figures and Tables

**Figure 1:**
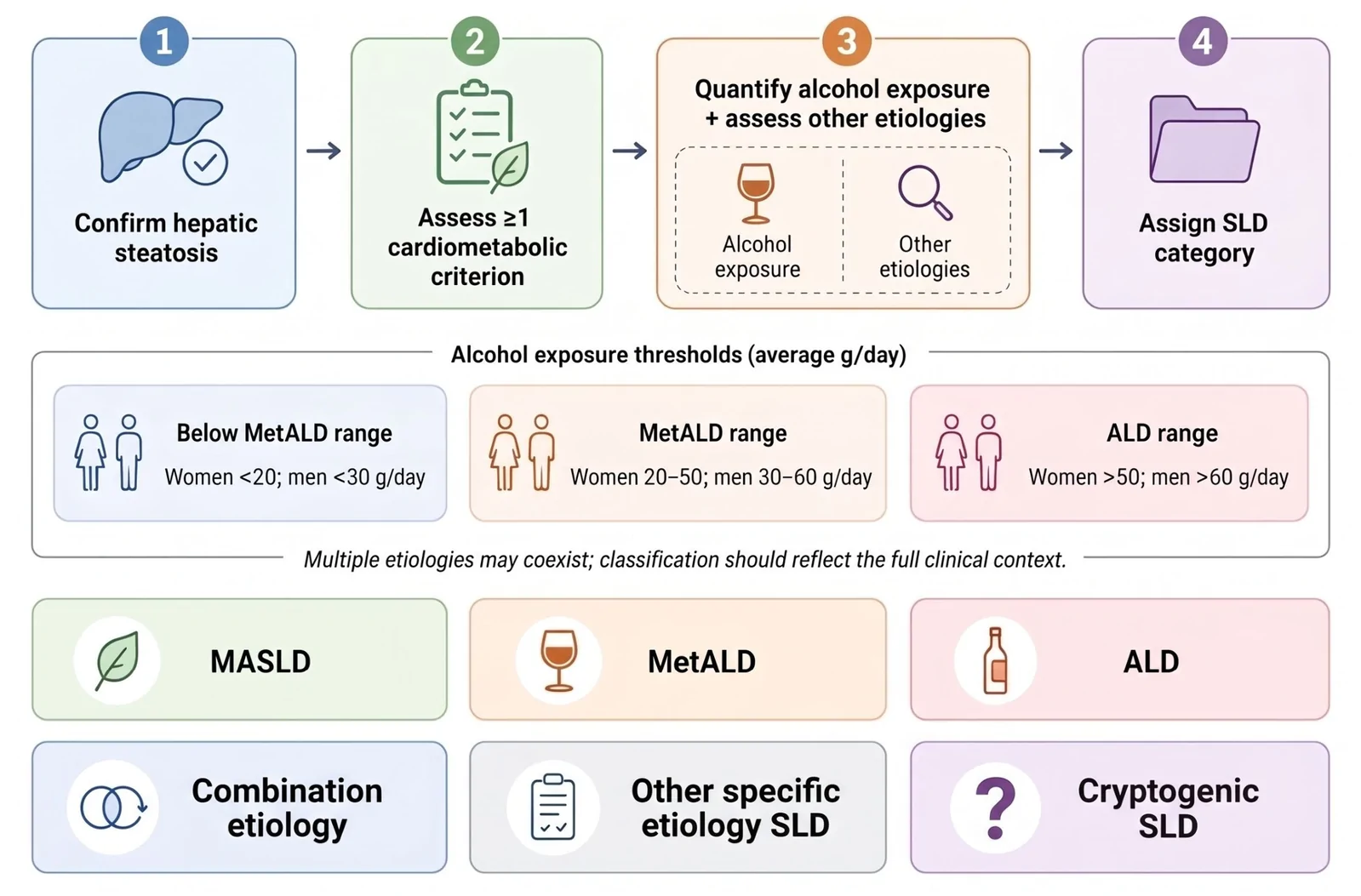
Practical framework for the classification of steatotic liver disease. This original schematic was prepared by the authors based on the nomenclature and diagnostic criteria proposed by Rinella et al. [4]. SLD, steatotic liver disease; MASLD, metabolic dysfunction-associated steatotic liver disease; MetALD, metabolic dysfunction-associated steatotic liver disease with increased alcohol consumption; ALD, alcohol-related liver disease; CMRF, cardiometabolic risk factor.

**Figure 3:**
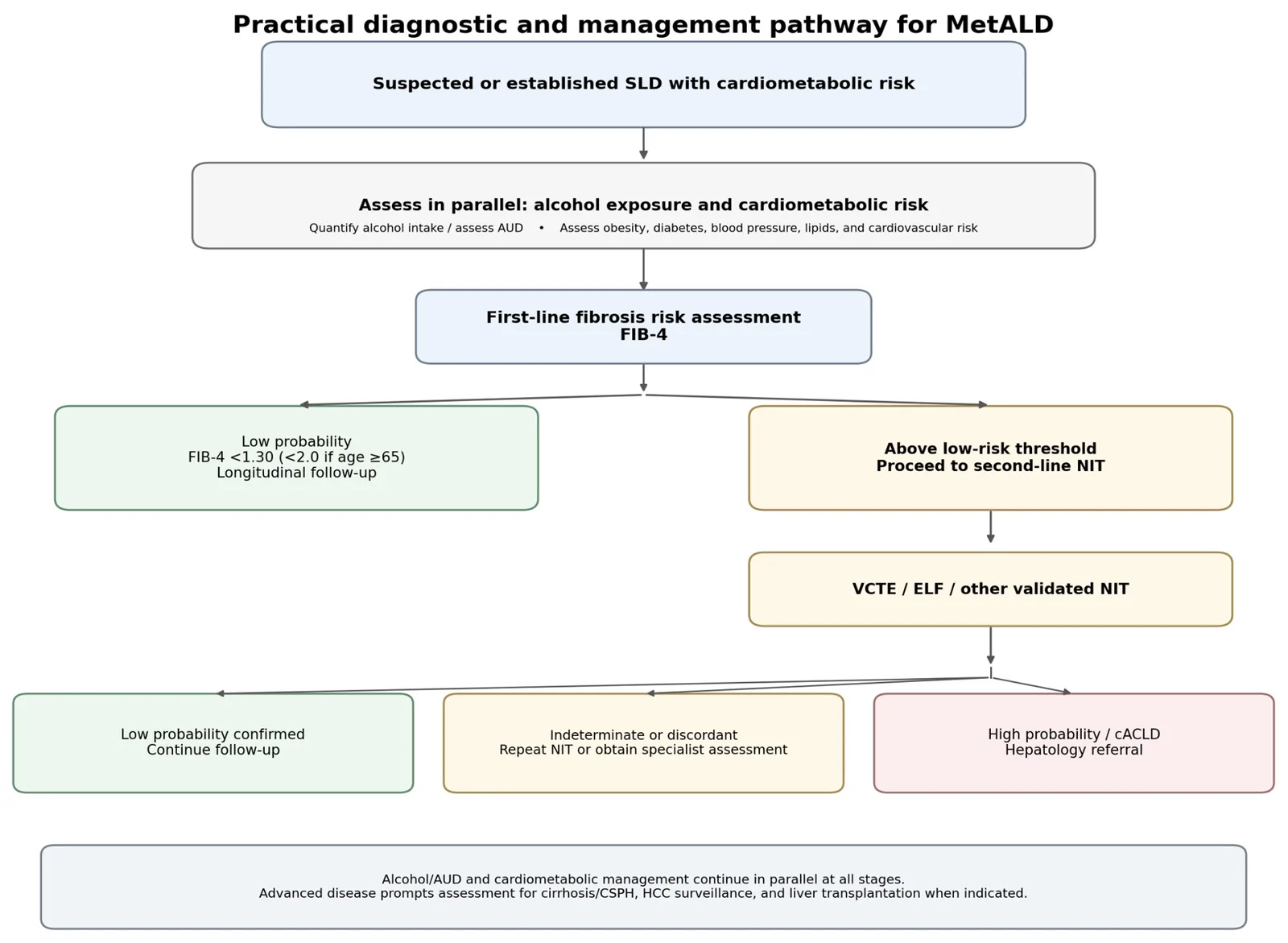
Practical diagnostic and management pathway for patients with suspected or established MetALD. Alcohol exposure and cardiometabolic risk should be assessed and managed in parallel. FIB-4 is used as a first-line fibrosis risk-stratification tool, followed by VCTE, ELF, or another validated second-line non-invasive test when the age-appropriate low-risk threshold is exceeded. Findings indicating a high probability of advanced fibrosis or cACLD warrant specialist evaluation and further assessment for cirrhosis and portal hypertension. Non-invasive thresholds are probabilistic and should be interpreted according to age, disease etiology, alcohol exposure, biochemical activity, and other potential confounding factors. Most thresholds currently applied in MetALD were originally derived in other chronic liver disease populations and have subsequently been evaluated to varying degrees in MetALD. This pathway represents an author-proposed synthesis of guideline-derived recommendations and the currently available MetALD-specific evidence; it has not been prospectively validated as a MetALD-specific management algorithm [5,39,40,50,54,69].

**Table 1:** Practical summary of cardiometabolic criteria used in the diagnosis of metabolic dysfunction-associated steatotic liver disease (MASLD).

Cardiometabolic Domain	Operational Criterion
Adults	
Adiposity	Body mass index (BMI) ≥ 25 kg/m^2^ (≥23 kg/m^2^ in Asian populations), increased waist circumference (>94 cm in men or >80 cm in women), or an ethnicity-adjusted equivalent.
Dysglycemia/type 2 diabetes	Fasting glucose ≥ 5.6 mmol/L (100 mg/dL), 2 h post-load glucose ≥ 7.8 mmol/L (140 mg/dL), HbA1c ≥ 5.7% (39 mmol/mol), diagnosed type 2 diabetes, or treatment for type 2 diabetes.
Blood pressure	Blood pressure ≥ 130/85 mmHg or antihypertensive treatment.
Triglycerides	Triglycerides ≥ 1.70 mmol/L (150 mg/dL) or lipid-lowering treatment.
High-density lipoprotein (HDL) cholesterol	HDL cholesterol ≤ 1.0 mmol/L (40 mg/dL) in men or ≤1.3 mmol/L (50 mg/dL) in women, or lipid-lowering treatment.
Children and adolescents	
Adiposity	BMI ≥ 85th percentile for age and sex (BMI z score ≥ +1), waist circumference >95th percentile, or an ethnicity-adjusted equivalent.
Dysglycemia/type 2 diabetes	Fasting glucose ≥ 5.6 mmol/L (100 mg/dL), random glucose ≥ 11.1 mmol/L (200 mg/dL), 2 h post-load glucose ≥ 7.8 mmol/L (140 mg/dL), HbA1c ≥ 5.7% (39 mmol/mol), diagnosed type 2 diabetes, or treatment for type 2 diabetes.
Blood pressure	Age < 13 years: blood pressure ≥95th percentile or ≥130/80 mmHg, whichever is lower; age ≥ 13 years: ≥130/85 mmHg; or antihypertensive treatment.
Triglycerides	Age < 10 years: triglycerides ≥1.15 mmol/L (100 mg/dL); age ≥ 10 years: ≥1.70 mmol/L (150 mg/dL); or lipid-lowering treatment.
HDL cholesterol	HDL cholesterol ≤ 1.0 mmol/L (40 mg/dL) or lipid-lowering treatment.

Meeting just one of these five cardiometabolic domains is enough to satisfy the metabolic criterion for MASLD in a patient with hepatic steatosis. The numerical thresholds follow the multisociety SLD nomenclature consensus and the current MASLD guideline [4,5]; the table’s layout and wording, however, were developed by the authors.

**Table 2:** VCTE and platelet count thresholds for advanced fibrosis, cACLD, CSPH, and variceal screening.

Clinical Purpose	Criterion	Practical Interpretation
Advanced fibrosis assessment	LSM < 8 kPa	Lower probability of advanced fibrosis
Advanced fibrosis assessment	LSM approximately 12–15 kPa or higher	Increasing probability of advanced fibrosis; interpret with clinical context and potential confounders
Identification of cACLD	LSM < 10 kPa	Low likelihood of cACLD when other clinical or imaging signs are absent
Identification of cACLD	LSM 10–15 kPa	Intermediate likelihood; further clinical and/or imaging assessment is appropriate
Identification of cACLD	LSM > 15 kPa	High likelihood of cACLD
Exclusion of CSPH	LSM ≤ 15 kPa and PLT ≥ 150 × 10^9^/L	CSPH can be excluded with high confidence in populations addressed by Baveno VII
Identification of CSPH	LSM ≥ 25 kPa	Supports CSPH in etiologies specified by Baveno VII; performance is less certain in obesity
Variceal screening when NSBB therapy is contraindicated or not tolerated	LSM < 20 kPa and PLT > 150 × 10^9^/L	Screening endoscopy can generally be deferred with continued non-invasive follow-up
Variceal screening when NSBB therapy is contraindicated or not tolerated	LSM ≥ 20 kPa or PLT ≤ 150 × 10^9^/L	Screening endoscopy is recommended

Abbreviations: cACLD, compensated advanced chronic liver disease; CSPH, clinically significant portal hypertension; LSM, liver stiffness measurement; NSBB, non-selective beta-blocker; PLT, platelet count; VCTE, vibration-controlled transient elastography. This table was compiled and reformulated by the authors from current EASL guidance [50] and the Baveno VII consensus [54]. Advanced fibrosis thresholds are based principally on EASL guidance, whereas cACLD, CSPH, and variceal-screening criteria are based on Baveno VII. These thresholds were not specifically derived in MetALD.

**Table 3:** Evidence supporting commonly used non-invasive tests and thresholds in MetALD and their principal limitations.

Non-Invasive Test/Threshold	Original Evidence Base	MetALD-Specific Evaluation	Clinical Role/Findings Relevant to MetALD	Principal Limitation
FIB-4 < 1.30 (<2.00 if age ≥ 65 years); ≥2.67	Developed and validated outside MetALD	Yes—Roy: *n* = 168; advanced fibrosis *n* = 31; MRE reference; retrospective. Gu: *n* = 369; biopsy *n* = 192; ≥F3 *n* = 44; histology; retrospective cross-sectional. Torp: *n* = 102; biopsy *n* = 78; ≥F3 *n* = 19; histology; prospective cohorts.	Torp: rule-out sensitivity 95% (95% CI 74–100%) and NPV 96% (80–100%); rule-in specificity 79% (66–88%). Roy: AUROC 0.78 (0.68–0.87) for MRE-defined advanced fibrosis. Gu: AUROC 0.773 in biopsy-proven MetALD [38,39,40].	Thresholds were not derived in MetALD; age-dependent performance; alcohol-related changes in aminotransferases and platelets may confound results.
APRI < 0.50/≥1.50	Developed in chronic hepatitis C	Limited	Roy: AUROC 0.58 (95% CI 0.46–0.70) in MetALD [38].	Inferior performance to FIB-4; AST and platelet count are susceptible to alcohol-related confounding.
ELF < 9.8/≥11.3	Established in chronic liver disease populations outside MetALD	Yes—Torp: *n* = 102; biopsy *n* = 78; ≥F3 *n* = 19; histology; prospective cohorts.	Torp: ELF < 9.8 sensitivity 100% (82–100%) and NPV 100% (91–100%); ELF ≥ 11.3 specificity 93% (84–98%) [40].	Single-cohort MetALD evidence and limited external validation; availability and cost vary.
VCTE/LSM	Extensive ALD, MASLD, viral, and mixed-etiology evidence	Yes—Gu: *n* = 369; biopsy *n* = 192; ≥F3 *n* = 44; histology; retrospective cross-sectional. Torp: *n* = 102; biopsy *n* = 78; ≥F3 *n* = 19; histology; prospective cohorts.	Gu: VCTE AUROC 0.833 in the biopsy-proven cohort; standard FIB-4→VCTE pathway false-negative rate 4.9% in MetALD. Torp also supported established VCTE-based pathways [39,40].	No independently derived MetALD-specific LSM thresholds; inflammation, recent alcohol use, cholestasis, congestion, and technical factors can alter LSM.
Baveno cACLD/CSPH criteria	Baveno VII and mixed chronic liver disease populations	No dedicated validation	LSM < 10/10–15/>15 kPa provides a general cACLD framework. CSPH rule-out: LSM ≤ 15 kPa + PLT ≥ 150 × 10^9^/L; rule-in: LSM ≥ 25 kPa in specified etiologies [54].	Not MetALD-specific; the ≥25 kPa rule-in criterion is not universally applicable in obesity.
CAP 248/268/280 dB/m	Individual-patient-data meta-analysis of mixed chronic liver disease	No dedicated validation	For ≥S1/≥S2/S3: cut-offs 248 (237–261), 268 (257–284), 280 (268–294) dB/m; AUROCs 0.823 (0.809–0.837), 0.865 (0.850–0.880), 0.882 (0.858–0.906); sensitivities 0.688 (0.600–0.750), 0.773 (0.690–0.838), 0.882 (0.765–0.956); specificities 0.822 (0.761–0.897), 0.812 (0.749–0.879), 0.776 (0.720–0.821) [55].	Predominantly viral cohort; thresholds influenced by BMI, diabetes, and etiology and should not be treated as precise histological boundaries.
FAST	Developed and validated in NAFLD/NASH	No dedicated validation	Combines LSM, CAP, and AST; original ≤0.35 and ≥0.67 thresholds were developed for rule-out/rule-in of fibrotic NASH [64].	No established MetALD-specific performance or optimal threshold.
Agile 3+/Agile 4	Developed and validated predominantly in NAFLD/MASLD	No dedicated validation	FibroScan (Echosens, Paris, France)-based scores combining LSM with clinical and laboratory variables improve identification of advanced fibrosis and cirrhosis in NAFLD/MASLD cohorts [65].	MetALD-specific validation remains insufficient.
FibroTest	Studied across chronic liver disease populations, including alcohol-related and metabolic liver disease [43].	Limited evidence available in the literature reviewed.	Proprietary serum fibrosis panel that may provide additional fibrosis assessment when simple serum-based scores are inconclusive [43].	Limited MetALD-specific evidence; no established MetALD-specific thresholds; cost and availability may limit routine use.
2D-SWE	Ultrasound elastography evidence across chronic liver disease populations [51,52].	No dedicated MetALD-specific validation identified in the literature reviewed.	Alternative ultrasound-based technique for liver stiffness assessment with real-time imaging guidance [51,52].	No established MetALD-specific cut-offs in the evidence reviewed; measurements may vary according to platform, technique, obesity, and clinical context.
MRE	Chronic liver disease and MASLD evidence [61,62,63].	Used as a reference standard in a MetALD study [38].	Highly accurate assessment of liver stiffness over a relatively large liver volume; useful when ultrasound-based methods are technically limited or discordant [61,62,63].	Cost and limited availability; MRI-related contraindications or practical limitations; signal loss may occur with marked iron overload [63].

Data were compiled by the authors from the cited original studies and clinical guidance documents. Values in parentheses are 95% confidence intervals unless otherwise stated. Abbreviations used in this table include AUROC, area under the receiver operating characteristic curve; NPV, negative predictive value.

**Table 4:** Pharmacological options for alcohol use disorder in patients with liver disease.

Drug	Clinical Role	Liver Disease Considerations	Renal Considerations	Practical Points
Baclofen	Option with cirrhosis-specific evidence	Among the best-studied options in cirrhosis; minimal hepatic metabolism	Predominantly renal elimination; caution/dose adjustment with renal impairment	Sedation and neurocognitive effects may be important in patients at risk of encephalopathy
Acamprosate	Established pharmacological option	Minimal hepatic metabolism	Dose reduction at creatinine clearance 30–50 mL/min; contraindicated at ≤30 mL/min	Assess renal function before treatment
Naltrexone	Established pharmacological option	Guideline-supported use best established in compensated disease; cirrhosis safety data are reassuring, but evidence in decompensated disease remains limited	Use cautiously in significant renal impairment	Contraindicated with opioid use or current opioid dependence
Gabapentin	Alternative option	Limited hepatotoxicity; less evidence in advanced liver disease	Dose adjustment required	Sedation may be clinically relevant
Topiramate	Alternative option	Limited cirrhosis-specific evidence	Dose reduction with renal impairment	Cognitive adverse effects may limit use
Disulfiram	Generally unsuitable in liver disease	Should generally be avoided in liver disease because of hepatotoxicity	Not the principal limiting factor	Risk of severe hepatic injury

Pharmacological treatment should be individualized according to liver disease severity, renal function, other medications, encephalopathy risk, psychiatric comorbidities, and patient preference. Evidence in MetALD specifically remains limited and is largely extrapolated from AUD and ALD studies. The table brings together the clinical evidence and guidance discussed in this section [70,71,72,73].

**Table 5:** Key randomized trials of incretin-based therapies relevant to MetALD.

Study	Population	Phase/Design and Sample Size	Intervention and Comparator	Main Finding	Relevance and Limitation for MetALD
ESSENCE	MASH, F2–F3	Phase 3; prespecified interim analysis, *n* = 800	Semaglutide 2.4 mg weekly vs. placebo	MASH resolution without fibrosis worsening: 62.9% vs. 34.3%; difference 28.7 pp (95% CI 21.1–36.2). Fibrosis improvement without MASH worsening: 36.8% vs. 22.4%; difference 14.4 pp (95% CI 7.5–21.3) [75].	Strong hepatic evidence, but not a MetALD trial.
SYNERGY-NASH	MASH, F2–F3	Phase 2; *n* = 190; 52 weeks	Tirzepatide 5, 10, or 15 mg weekly vs. placebo	MASH resolution: 44%, 56%, and 62% with 5, 10, and 15 mg, respectively, vs. 10% with placebo. Fibrosis improvement without worsening of MASH: 55%, 51%, and 51%, respectively, vs. 30% with placebo; differences 25 pp (95% CI 5–46), 22 pp (95% CI 1–42), and 21 pp (95% CI 1–42), respectively [76].	Histological MASH evidence; not MetALD.
Retatrutide MASLD substudy	MASLD with increased liver fat	Phase 2a; *n* = 98	Retatrutide 1, 4, 8, or 12 mg vs. placebo	At 24 weeks, 12 mg reduced liver fat by 82.4% vs. a 0.3% increase with placebo; treatment difference −82.7% (95% CI −95.2 to −70.2; *p* < 0.001) [77].	MRI-PDFF endpoint; no histological MASH/fibrosis endpoint; not MetALD.
Hendershot et al. [78]	AUD	Phase 2; *n* = 48; 9 weeks	Low-dose semaglutide vs. placebo	Laboratory alcohol self-administration (grams of alcohol consumed): standardized β −0.48 (95% CI −0.85 to −0.11); drinks per drinking day: standardized β −0.41 (95% CI −0.73 to −0.09); weekly alcohol craving (Penn Alcohol Craving Scale, 0–30): standardized β −0.39 (95% CI −0.73 to −0.06) [78].	AUD outcomes; no liver-disease efficacy endpoint.
Klausen et al. [79]	AUD + obesity	Phase 2; *n* = 108; 26 weeks	Semaglutide 2.4 mg + CBT vs. placebo + CBT	Heavy drinking days decreased by 41.1 pp from baseline (95% CI −48.7 to −33.5) with semaglutide and by 26.4 pp (95% CI −34.1 to −18.6) with placebo; baseline-adjusted ANCOVA-estimated treatment difference −13.7 pp (95% CI −22.0 to −5.4; *p* = 0.0015) [79].	AUD trial; no MetALD-specific hepatic assessment.

Data were compiled by the authors from the cited randomized trials. None of these trials was conducted specifically in patients with MetALD; hepatic and alcohol-related outcomes were evaluated in separate patient populations. Abbreviations: AUD, alcohol use disorder; CBT, cognitive behavioral therapy; MASH, metabolic dysfunction-associated steatohepatitis; MASLD, metabolic dysfunction-associated steatotic liver disease; MRI-PDFF, magnetic resonance imaging proton density fat fraction; pp, percentage points.

## Data Availability

No new data were created or analyzed in this study. Data sharing is not applicable to this article.

## Abbreviations

2D-SWE	two-dimensional shear wave elastography
ALD	alcohol-related liver disease
ALT	alanine aminotransferase
APRI	AST-to-Platelet Ratio Index
AST	aspartate aminotransferase
AUD	alcohol use disorder
AUDIT	Alcohol Use Disorders Identification Test
AUDIT-C	Alcohol Use Disorders Identification Test–Consumption
AUROC	area under the receiver operating characteristic curve
BMI	body mass index
cACLD	compensated advanced chronic liver disease
CAP	controlled attenuation parameter
CBT	cognitive behavioral therapy
CI	confidence interval
CSPH	clinically significant portal hypertension
DNL	de novo lipogenesis
ELF	Enhanced Liver Fibrosis
EtG	ethyl glucuronide
EtS	ethyl sulfate
FAST	FibroScan-AST
FFA	free fatty acid
FIB-4	Fibrosis-4 index
FMT	fecal microbiota transplantation
GGT	gamma-glutamyl transferase
GIP	glucose-dependent insulinotropic polypeptide
GLP-1	glucagon-like peptide-1
HCC	hepatocellular carcinoma
HDL	high-density lipoprotein
HRS-AKI	hepatorenal syndrome–acute kidney injury
IQR/M	interquartile range-to-median ratio
LPS	lipopolysaccharide
LSM	liver stiffness measurement
LT	liver transplantation
MASH	metabolic dysfunction-associated steatohepatitis
MASLD	metabolic dysfunction-associated steatotic liver disease
MELD	Model for End-Stage Liver Disease
MetALD	metabolic dysfunction-associated steatotic liver disease with increased alcohol consumption
MRE	magnetic resonance elastography
MRI-PDFF	magnetic resonance imaging proton density fat fraction
NAFLD	non-alcoholic fatty liver disease
NASH	non-alcoholic steatohepatitis
NIT	non-invasive test
NPV	negative predictive value
NSBB	non-selective beta-blocker
PAMPs	pathogen-associated molecular patterns
PEth	phosphatidylethanol
PLT	platelet count
ROS	reactive oxygen species
SLD	steatotic liver disease
TLR4	Toll-like receptor 4
ULN	upper limit of normal
VCTE	vibration-controlled transient elastography
